# 3-Hy­droxy-4-(3-hy­droxy­phen­yl)-2-quinolone monohydrate

**DOI:** 10.1107/S160053681102945X

**Published:** 2011-07-30

**Authors:** Yi-Wen Tao, Yun Wang

**Affiliations:** a School of Basic Science, Guangzhou Medical College, Guangzhou 510182, People’s Republic of China; b Guangdong Institute for Drug Control, Guangzhou 510180, People’s Republic of China

## Abstract

In the title compound, also known as viridicatol monohydrate, C_15_H_11_NO_3_·H_2_O, the dihedral angle between the benzene ring and quinoline ring system is 64.76 (5)°. An intra­molecular O—H⋯O hydrogen bond occurs. The crystal structure is stabilized by classical inter­molecular N—H⋯O and O—H⋯O hydrogen bonds and weak π–π inter­actions with a centroid–centroid distance of 3.8158 (10) Å.

## Related literature

For 3-hy­droxy-2(1*H*)-pyridinone, see: Deflon *et al.* (2000 ▶) and for 3-hy­droxy-2-oxo-1,2-dihydro­quinoline, see: Strashnova *et al.* (2008 ▶). For the isolation of viridicatol, see: Yurchenko *et al.* (2010 ▶); Fremlin *et al.* (2009 ▶); Proksch *et al.* (2008 ▶); Lund & Frisvad (1994 ▶); Birkinshaw *et al.* (1963 ▶); Kozlovskii *et al.* (2002 ▶). For the synthesis of viridicatol, see: Kobayashi & Harayama (2009 ▶). For examples of viridicatol derivatives, see: Bracken *et al.* (1954 ▶). For the biological activity of viridicatol, see: Lin *et al.* (2008 ▶); Proksch *et al.* (2008 ▶). For a description of the Cambridge Structural Database, see: Allen (2002 ▶).
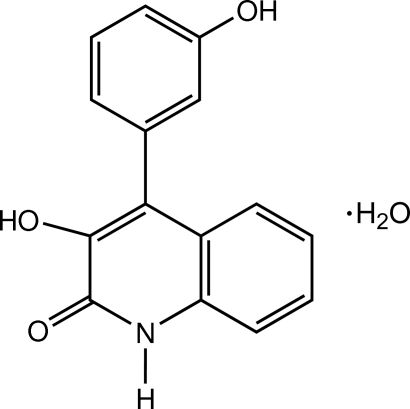


## Experimental

### 

#### Crystal data

C_15_H_11_NO_3_·H_2_O
*M* *_r_* = 271.26
Triclinic, 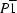

*a* = 6.9845 (5) Å
*b* = 10.0632 (7) Å
*c* = 10.3361 (6) Å
α = 109.204 (6)°
β = 103.251 (5)°
γ = 101.015 (6)°
*V* = 639.12 (9) Å^3^
*Z* = 2
Cu *K*α radiation
μ = 0.86 mm^−1^
*T* = 298 K
0.30 × 0.20 × 0.05 mm

#### Data collection

Bruker SMART CCD area-detector diffractometer
Absorption correction: multi-scan (*SADABS*; Bruker, 2002 ▶) *T*_min_ = 0.783, *T*_max_ = 0.958
5057 measured reflections
2225 independent reflections
1958 reflections with *I* > 2σ(*I*)
*R*_int_ = 0.018

#### Refinement

*R*[*F* ^2^ > 2σ(*F* ^2^)] = 0.042
*wR*(*F* ^2^) = 0.132
*S* = 1.10
2225 reflections
184 parameters
1 restraint
H-atom parameters constrained
Δρ_max_ = 0.22 e Å^−3^
Δρ_min_ = −0.27 e Å^−3^

### 

Data collection: *SMART* (Bruker, 2002 ▶); cell refinement: *SAINT* (Bruker, 2002 ▶); data reduction: *SAINT*; program(s) used to solve structure: *SHELXS97* (Sheldrick, 2008 ▶); program(s) used to refine structure: *SHELXL97* (Sheldrick, 2008 ▶); molecular graphics: *ORTEP-3 for Windows* (Farrugia, 1997 ▶); software used to prepare material for publication: *WinGX* (Farrugia, 1999 ▶).

## Supplementary Material

Crystal structure: contains datablock(s) I, global. DOI: 10.1107/S160053681102945X/bg2408sup1.cif

Structure factors: contains datablock(s) I. DOI: 10.1107/S160053681102945X/bg2408Isup2.hkl

Supplementary material file. DOI: 10.1107/S160053681102945X/bg2408Isup3.mol

Supplementary material file. DOI: 10.1107/S160053681102945X/bg2408Isup4.cml

Additional supplementary materials:  crystallographic information; 3D view; checkCIF report

## Figures and Tables

**Table 1:** Hydrogen-bond geometry (Å, °)

*D*—H⋯*A*	*D*—H	H⋯*A*	*D*⋯*A*	*D*—H⋯*A*
N1—H1*A*⋯O1^i^	0.86	2.11	2.9577 (16)	169
O1—H1⋯O1*W*	0.82	1.92	2.689 (2)	155
O2—H2*A*⋯O3^ii^	0.82	1.99	2.6500 (16)	138
O2—H2*A*⋯O3	0.82	2.28	2.7242 (14)	115
O1*W*—H1*B*⋯O1*W*^iii^	0.86	2.37	2.816 (3)	113
O1*W*—H1*C*⋯O2^iv^	0.86	2.04	2.8476 (18)	157

Symmetry codes: (i) 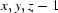; (ii) 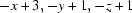; (iii) 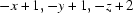; (iv) 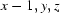.

**Fig. 1.**
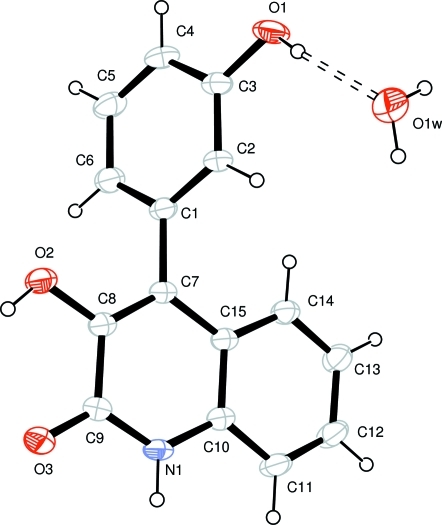
The molecular structure of (I), with atom labels and 30% probability displacement ellipsoids for non-H atoms. The hydrogen bonds are shown as dashed lines.

## supplementary crystallographic information

### Comment

3-hydroxy-4-(3-hydroxyphenyl)-2-quinolone, also known as viridicatol, C_15_H_13_NO_4_ (I), was first isolated from Penicillium viridicatum Westling (Birkinshaw *et al.*, 1963). It has been proven that viridicatol can completely inhibit the spleen lymphocytes proliferation (Lin *et al.*, 2008) and suppress larval growth of the polyphagous pest insect Spodoptera littoralis (Proksch *et al.*, 2008). Viridicatol can be isolated from Penicillium aurantiogriseum sensu lato (Lund *et al.*, 1994), Penicillium chrysogenum strains (Kozlovskii *et al.*, 2002), Penicillium *sp* from specimens of suberites domuncula (Proksch *et al.*, 2008), an Australian marine-derived isolate of Aspergillus versicolor (Fremlin *et al.*, 2009), and the marine fungus Aspergillus versicolor (Yurchenko *et al.*, 2010). A similar compound, viridicatin, could be isolated from Penicillium cyclopium Westling (Bracken *et al.*, 1954). Viridicatol also can be synthesized by one-pot method from cyanoacetanilides through Knoevenagel condensation followed by decyanative epoxide-arene cyclization (Kobayashi *et al.*, 2009), but so far the crystal structure of viridicatol has not been reported.

The title compound can be considered as containing embedded 3-hydroxy-2(1*H*)-pyridinone, or 3-hydroxy-2-oxo-1,2-dihydroquinoline, motifs (Fig. 1). The crystal structures of both groups have already been reported (Deflon *et al.*, 2000 and  Strashnova *et al.*, 2008)  and their structural parameters are similar to those in I.

The 3-hydroxylbenzyl ring subtends a torsion angle of 64.76 (5)° to the quinoline to reduce the steric effect. The structure contains a water molecule which is involved in three out of the six  hydrogen bonds formed (Table 1). The whole structure is a 3-D hydrogen-bonding architecture, futther stabilized by the weak π-π interaction between two pyridinone rings with a *Cg*1···*Cg*1 (2 - *x*,1 - *y*,1 - *z*) separation of 3.8158 (10) Å and the dihedral angle is zero (where *Cg*1 is the centroid of the N1/C7—C10/C15). Both weak interactions of hydrogen bonds and π-π effect consolidate the stability of the structure.

### Experimental

The fungus phomposis *sp* was isolated from the mangrove tree, Zhanjiang, and was stored at the Department of Applied Chemistry, Zhongshan University, Guangzhou, China. Starter cultures (from Professor Shining Zhou) were maintained on cornmeal seawater agar. Plugs of agar supporting mycelium growth were cut from solid culture medium and transferred aseptically to a 250 ml Erlenmeyer flask containing 100 ml liquid medium. The fungus was incubated at 28 °C and placed thirty days. The culture was filtered through cheesecloth. The mycelium was air-dried and then extracted in methanol. The CH_3_OH extract of the fungal mycelium was chromatographed on silica gel by using a gradient from petroleum to ethyl acetate, then from acetate to methanol, and obtained viridicatol eluted with 50% ethyl acetate-petroleum ether. Colorless block crystals were grown from a solution in methanol at room temperature over several days.

### Refinement

H atoms bonded to C atoms were positioned geometrically and treated as riding, with C—H distances of 0.93 Å and *U*_iso_(H)=1.2*U*_eq_(C). H atoms involved in hydrogen-bonding interactions (water, pyridinone, and hydroxy) were located from difference Fourier maps, idealized and refined with a riding scheme.

### Crystal data

**Table:**

C_15_H_11_NO_3_·H_2_O	*Z* = 2
*M**_r_* = 271.26	*F*(000) = 284
Triclinic, *P*1	*D*_x_ = 1.410 Mg m^−^^3^
Hall symbol: -P 1	Cu *K*α radiation, λ = 1.54178 Å
*a* = 6.9845 (5) Å	Cell parameters from 5194 reflections
*b* = 10.0632 (7) Å	θ = 4.8–69.4°
*c* = 10.3361 (6) Å	µ = 0.86 mm^−^^1^
α = 109.204 (6)°	*T* = 298 K
β = 103.251 (5)°	Block, colorless
γ = 101.015 (6)°	0.30 × 0.20 × 0.05 mm
*V* = 639.12 (9) Å^3^	

### Data collection

**Table:**

Bruker SMART CCD area-detector diffractometer	2225 independent reflections
Radiation source: fine-focus sealed tube	1958 reflections with *I* > 2σ(*I*)
graphite	*R*_int_ = 0.018
φ and ω scans	θ_max_ = 66.0°, θ_min_ = 4.8°
Absorption correction: multi-scan (*SADABS*; Bruker, 2002)	*h* = −8→8
*T*_min_ = 0.783, *T*_max_ = 0.958	*k* = −11→11
5057 measured reflections	*l* = −12→11

### Refinement

**Table:**

Refinement on *F*^2^	Secondary atom site location: difference Fourier map
Least-squares matrix: full	Hydrogen site location: inferred from neighbouring sites
*R*[*F*^2^ > 2σ(*F*^2^)] = 0.042	H-atom parameters constrained
*wR*(*F*^2^) = 0.132	*w* = 1/[σ^2^(*F*_o_^2^) + (0.0774*P*)^2^ + 0.123*P*] where *P* = (*F*_o_^2^ + 2*F*_c_^2^)/3
*S* = 1.10	(Δ/σ)_max_ < 0.001
2225 reflections	Δρ_max_ = 0.22 e Å^−^^3^
184 parameters	Δρ_min_ = −0.27 e Å^−^^3^
1 restraint	Extinction correction: *SHELXL97* (Sheldrick, 2008), Fc^*^=kFc[1+0.001xFc^2^λ^3^/sin(2θ)]^-1/4^
Primary atom site location: structure-invariant direct methods	Extinction coefficient: 0.028 (3)

### Special details

**Table:**

Geometry. All esds (except the esd in the dihedral angle between two l.s. planes) are estimated using the full covariance matrix. The cell esds are taken into account individually in the estimation of esds in distances, angles and torsion angles; correlations between esds in cell parameters are only used when they are defined by crystal symmetry. An approximate (isotropic) treatment of cell esds is used for estimating esds involving l.s. planes.
Refinement. Refinement of *F*^2^ against ALL reflections. The weighted *R*-factor *wR* and goodness of fit *S* are based on *F*^2^, conventional *R*-factors *R* are based on *F*, with *F* set to zero for negative *F*^2^. The threshold expression of *F*^2^ > σ(*F*^2^) is used only for calculating *R*-factors(gt) *etc*. and is not relevant to the choice of reflections for refinement. *R*-factors based on *F*^2^ are statistically about twice as large as those based on *F*, and *R*- factors based on ALL data will be even larger.

### Fractional atomic coordinates and isotropic or equivalent isotropic displacement parameters (Å^2^)

**Table:**

	*x*	*y*	*z*	*U*_iso_*/*U*_eq_	
C1	1.0143 (2)	0.24292 (15)	0.66031 (14)	0.0370 (4)	
C2	0.9196 (2)	0.30457 (16)	0.75933 (15)	0.0385 (4)	
H2	0.8371	0.3630	0.7413	0.046*	
C3	0.9479 (2)	0.27913 (17)	0.88547 (15)	0.0428 (4)	
C4	1.0709 (3)	0.19305 (19)	0.91275 (17)	0.0516 (4)	
H4	1.0906	0.1766	0.9976	0.062*	
C5	1.1646 (3)	0.1315 (2)	0.81423 (18)	0.0542 (4)	
H5	1.2471	0.0732	0.8328	0.065*	
C6	1.1371 (3)	0.15549 (18)	0.68768 (17)	0.0474 (4)	
H6	1.2004	0.1133	0.6213	0.057*	
C7	0.9865 (2)	0.27142 (15)	0.52515 (14)	0.0359 (4)	
C8	1.1501 (2)	0.34609 (16)	0.50295 (15)	0.0402 (4)	
C9	1.1342 (2)	0.37555 (17)	0.37236 (16)	0.0401 (4)	
C10	0.7706 (2)	0.24388 (16)	0.28851 (15)	0.0386 (4)	
C11	0.5843 (3)	0.18891 (19)	0.17754 (17)	0.0504 (4)	
H11	0.5750	0.2066	0.0938	0.061*	
C12	0.4149 (3)	0.1087 (2)	0.19222 (19)	0.0565 (5)	
H12	0.2913	0.0699	0.1171	0.068*	
C13	0.4255 (3)	0.0846 (2)	0.31846 (19)	0.0533 (4)	
H13	0.3088	0.0321	0.3285	0.064*	
C14	0.6089 (2)	0.13873 (17)	0.42801 (17)	0.0439 (4)	
H14	0.6150	0.1220	0.5119	0.053*	
C15	0.7871 (2)	0.21856 (15)	0.41627 (14)	0.0361 (4)	
N1	0.94503 (19)	0.32206 (14)	0.27371 (13)	0.0416 (3)	
H1A	0.9317	0.3377	0.1956	0.050*	
O1	0.8573 (2)	0.33744 (15)	0.98597 (12)	0.0589 (4)	
H1	0.8083	0.3993	0.9673	0.088*	
O2	1.33807 (17)	0.39862 (15)	0.60257 (12)	0.0576 (4)	
H2A	1.4195	0.4438	0.5749	0.086*	
O3	1.28439 (17)	0.44529 (14)	0.35328 (12)	0.0537 (4)	
O1W	0.5902 (2)	0.4697 (2)	0.88892 (15)	0.0819 (5)	
H1B	0.4875	0.4143	0.8981	0.098*	
H1C	0.5456	0.4629	0.8014	0.098*	

### Atomic displacement parameters (Å^2^)

**Table:**

	*U*^11^	*U*^22^	*U*^33^	*U*^12^	*U*^13^	*U*^23^
C1	0.0379 (7)	0.0408 (7)	0.0291 (7)	0.0062 (6)	0.0054 (6)	0.0162 (6)
C2	0.0447 (8)	0.0449 (8)	0.0297 (7)	0.0136 (6)	0.0100 (6)	0.0201 (6)
C3	0.0505 (8)	0.0485 (8)	0.0297 (7)	0.0105 (7)	0.0108 (6)	0.0189 (6)
C4	0.0659 (10)	0.0592 (10)	0.0345 (8)	0.0197 (8)	0.0084 (7)	0.0283 (7)
C5	0.0641 (10)	0.0588 (10)	0.0450 (9)	0.0281 (8)	0.0084 (8)	0.0273 (8)
C6	0.0526 (9)	0.0534 (9)	0.0385 (8)	0.0207 (7)	0.0122 (7)	0.0192 (7)
C7	0.0408 (8)	0.0397 (7)	0.0283 (7)	0.0117 (6)	0.0106 (6)	0.0149 (6)
C8	0.0399 (8)	0.0484 (8)	0.0318 (8)	0.0104 (6)	0.0089 (6)	0.0180 (6)
C9	0.0432 (8)	0.0470 (8)	0.0349 (8)	0.0134 (6)	0.0149 (6)	0.0202 (6)
C10	0.0431 (8)	0.0422 (8)	0.0326 (7)	0.0134 (6)	0.0102 (6)	0.0178 (6)
C11	0.0523 (9)	0.0611 (10)	0.0385 (8)	0.0127 (8)	0.0041 (7)	0.0293 (8)
C12	0.0443 (9)	0.0672 (11)	0.0508 (10)	0.0068 (8)	−0.0037 (7)	0.0322 (8)
C13	0.0419 (8)	0.0615 (10)	0.0557 (10)	0.0062 (7)	0.0067 (7)	0.0327 (8)
C14	0.0438 (8)	0.0511 (8)	0.0395 (8)	0.0101 (7)	0.0104 (6)	0.0249 (7)
C15	0.0422 (8)	0.0385 (7)	0.0297 (7)	0.0134 (6)	0.0106 (6)	0.0156 (6)
N1	0.0471 (7)	0.0536 (7)	0.0300 (6)	0.0130 (6)	0.0121 (5)	0.0244 (6)
O1	0.0812 (9)	0.0802 (9)	0.0369 (6)	0.0369 (7)	0.0290 (6)	0.0349 (6)
O2	0.0408 (6)	0.0858 (9)	0.0426 (6)	−0.0010 (6)	0.0040 (5)	0.0379 (6)
O3	0.0456 (6)	0.0742 (8)	0.0509 (7)	0.0099 (6)	0.0170 (5)	0.0392 (6)
O1W	0.0676 (9)	0.1210 (13)	0.0514 (8)	0.0389 (9)	0.0136 (7)	0.0234 (8)

### Geometric parameters (Å, °)

**Table:**

C1—C2	1.385 (2)	C9—N1	1.351 (2)
C1—C6	1.389 (2)	C10—N1	1.3873 (19)
C1—C7	1.4941 (19)	C10—C11	1.392 (2)
C2—C3	1.388 (2)	C10—C15	1.409 (2)
C2—H2	0.9300	C11—C12	1.368 (2)
C3—O1	1.3659 (19)	C11—H11	0.9300
C3—C4	1.379 (2)	C12—C13	1.392 (2)
C4—C5	1.377 (3)	C12—H12	0.9300
C4—H4	0.9300	C13—C14	1.372 (2)
C5—C6	1.385 (2)	C13—H13	0.9300
C5—H5	0.9300	C14—C15	1.400 (2)
C6—H6	0.9300	C14—H14	0.9300
C7—C8	1.352 (2)	N1—H1A	0.8600
C7—C15	1.447 (2)	O1—H1	0.8200
C8—O2	1.3492 (18)	O2—H2A	0.8200
C8—C9	1.459 (2)	O1W—H1B	0.8646
C9—O3	1.2393 (19)	O1W—H1C	0.8616
			
C2—C1—C6	119.79 (13)	N1—C9—C8	115.61 (13)
C2—C1—C7	120.22 (13)	N1—C10—C11	120.39 (13)
C6—C1—C7	119.99 (13)	N1—C10—C15	118.72 (13)
C1—C2—C3	119.98 (14)	C11—C10—C15	120.87 (14)
C1—C2—H2	120.0	C12—C11—C10	119.70 (14)
C3—C2—H2	120.0	C12—C11—H11	120.1
O1—C3—C4	117.93 (13)	C10—C11—H11	120.2
O1—C3—C2	121.98 (14)	C11—C12—C13	120.70 (15)
C4—C3—C2	120.09 (15)	C11—C12—H12	119.6
C5—C4—C3	119.96 (14)	C13—C12—H12	119.6
C5—C4—H4	120.0	C14—C13—C12	119.69 (15)
C3—C4—H4	120.0	C14—C13—H13	120.2
C4—C5—C6	120.54 (15)	C12—C13—H13	120.2
C4—C5—H5	119.7	C13—C14—C15	121.50 (14)
C6—C5—H5	119.7	C13—C14—H14	119.3
C5—C6—C1	119.64 (15)	C15—C14—H14	119.3
C5—C6—H6	120.2	C14—C15—C10	117.51 (13)
C1—C6—H6	120.2	C14—C15—C7	123.74 (13)
C8—C7—C15	119.29 (13)	C10—C15—C7	118.71 (13)
C8—C7—C1	119.73 (13)	C9—N1—C10	125.12 (12)
C15—C7—C1	120.97 (13)	C9—N1—H1A	117.4
O2—C8—C7	121.09 (13)	C10—N1—H1A	117.4
O2—C8—C9	116.39 (13)	C3—O1—H1	109.5
C7—C8—C9	122.52 (14)	C8—O2—H2A	109.5
O3—C9—N1	122.18 (13)	H1B—O1W—H1C	103.4
O3—C9—C8	122.21 (14)		
			
C6—C1—C2—C3	0.1 (2)	C7—C8—C9—N1	−0.6 (2)
C7—C1—C2—C3	−179.25 (13)	N1—C10—C11—C12	178.30 (15)
C1—C2—C3—O1	−179.90 (14)	C15—C10—C11—C12	−0.2 (3)
C1—C2—C3—C4	0.3 (2)	C10—C11—C12—C13	1.6 (3)
O1—C3—C4—C5	179.72 (15)	C11—C12—C13—C14	−1.6 (3)
C2—C3—C4—C5	−0.5 (3)	C12—C13—C14—C15	0.2 (3)
C3—C4—C5—C6	0.2 (3)	C13—C14—C15—C10	1.1 (2)
C4—C5—C6—C1	0.2 (3)	C13—C14—C15—C7	−176.67 (15)
C2—C1—C6—C5	−0.4 (2)	N1—C10—C15—C14	−179.69 (13)
C7—C1—C6—C5	179.01 (15)	C11—C10—C15—C14	−1.1 (2)
C2—C1—C7—C8	115.56 (16)	N1—C10—C15—C7	−1.8 (2)
C6—C1—C7—C8	−63.8 (2)	C11—C10—C15—C7	176.81 (14)
C2—C1—C7—C15	−65.28 (19)	C8—C7—C15—C14	179.18 (14)
C6—C1—C7—C15	115.34 (16)	C1—C7—C15—C14	0.0 (2)
C15—C7—C8—O2	179.24 (13)	C8—C7—C15—C10	1.4 (2)
C1—C7—C8—O2	−1.6 (2)	C1—C7—C15—C10	−177.78 (12)
C15—C7—C8—C9	−0.2 (2)	O3—C9—N1—C10	−179.68 (14)
C1—C7—C8—C9	178.97 (13)	C8—C9—N1—C10	0.2 (2)
O2—C8—C9—O3	−0.2 (2)	C11—C10—N1—C9	−177.58 (15)
C7—C8—C9—O3	179.28 (15)	C15—C10—N1—C9	1.0 (2)
O2—C8—C9—N1	179.94 (13)		

### Hydrogen-bond geometry (Å, °)

**Table:**

*D*—H···*A*	*D*—H	H···*A*	*D*···*A*	*D*—H···*A*
N1—H1A···O1^i^	0.86	2.11	2.9577 (16)	169.
O1—H1···O1W	0.82	1.92	2.689 (2)	155.
O2—H2A···O3^ii^	0.82	1.99	2.6500 (16)	138.
O2—H2A···O3	0.82	2.28	2.7242 (14)	115.
O1W—H1B···O1W^iii^	0.86	2.37	2.816 (3)	113.
O1W—H1C···O2^iv^	0.86	2.04	2.8476 (18)	157.

Symmetry codes: (i) *x*, *y*, *z*−1; (ii) −*x*+3, −*y*+1, −*z*+1; (iii) −*x*+1, −*y*+1, −*z*+2; (iv) *x*−1, *y*, *z*.
